# Evidence of Oropharyngeal Dysfunction in Feeding in the Rat Rotenone Model of Parkinson's Disease

**DOI:** 10.1155/2018/6537072

**Published:** 2018-03-11

**Authors:** François D. H. Gould, Andrew Gross, Rebecca Z. German, Jason R. Richardson

**Affiliations:** ^1^Department of Anatomy and Neurobiology, Northeast Ohio Medical University, Rootstown, OH, USA; ^2^Department of Pharmaceutical Science, Northeast Ohio Medical University, Rootstown, OH, USA

## Abstract

Swallowing disorders in Parkinson's disease are not responsive to dopamine depletion therapy and contribute to morbidity. They are poorly understood owing to a lack of adequate models. We present the first evidence of oropharyngeal changes in a rotenone toxicity model of Parkinson's disease. Rats were recorded while feeding before and after daily rotenone injections at two different doses (2.75 mg/kg and 3 mg/kg). The higher dose had a much more severe parkinsonian phenotype than the low dose. Timing and amplitude of chewing changed, as did the coordination of chewing and swallowing. Dose-dependent effects were evident. These preliminary results indicate that future research in toxicological models of Parkinson's disease should incorporate the study of oropharyngeal dysfunction. A better understanding of nongenetic models of Parkinson's disease in feeding may open new avenues for research into the neurological and behavioral bases for swallowing dysfunction in Parkinson's disease.

## 1. Introduction

Parkinson's disease is the second most common primary neurodegenerative disorder worldwide, with both genetic and environmental etiologies [1–3]. The characteristic signs of Parkinson's disease—bradykinesia, rigidity, and tremor—have been linked to dopamine loss resulting from neuronal death in the substantia nigra [2, 4, 5]. However, it is becoming clear that damage occurs on a larger scale, throughout the nervous system in PD, and that many symptoms are not responsive to treatments targeting dopamine loss [6], including problems with eating and swallowing [7, 8]. Such problems are frequent in patients with Parkinson's disease. In one study, 68% of late-stage Parkinson's disease patients reported subjective dysphagia symptoms [9]. Using objective diagnostic tools such as videofluoroscopy, another study reported that over half the early stage patients in the study had objective signs of dysphagia without reporting symptoms [10]. Feeding parameters respond inconsistently to current treatments for Parkinson's diseases, deep brain stimulation, and L-dopa. Certain aspects may show improvement while others do not within the same study [11]. In other studies, responses do not improve [12, 13] or even get worse [14] with L-dopa or deep brain stimulation. As well as presenting a challenge for effective, consistent treatment of dysphagia in Parkinson's disease, these conflicting results indicate that the neurological basis for dysphagia in Parkinson's disease is probably complex and poorly understood. These significant problems constitute a major medical challenge for PD patients, severely reducing the quality of life [15]. Further, aspiration pneumonia, a complication of chronic dysphagia (pathological swallowing), has an incidence rate four times that of the general population in Parkinson's disease [16, 17] and is the leading cause of death in long-term studies [18]. The etiology, progression, and neurological basis of swallowing dysfunction in PD remain poorly understood [19, 20], owing in part to the lack of animal models addressing this question specifically [21].

The complex 1 inhibitor rotenone, a commercially available pesticide, has become established as a compound which, when administered to rats, reproduces many of the neurodegenerative and behavioral phenotypes of Parkinson's disease [22–24]. These include nigrostriatal dopamine loss, alpha synuclein aggregation, polyubiquitin formation, and evidence of inflammation and microglial activation. Behaviorally, rats with rotenone-induced parkinsonism display rigidity, bradykinesia, reduced mobility, and reduced performance in a number of tasks associated with locomotion and limb use. However, the effect of rotenone injection on swallowing and feeding function has not been assessed. In fact, so far only very limited animal model work has focused on the swallowing dysfunction component of Parkinson's disease, including studies on a single genetic variant, the Pink1 rat [25, 26], and some work on chewing in nonhuman primate models. Work on the Pink1 rat has indicated that oropharyngeal dysfunction in Parkinson's disease may appear as an early onset sign [25]; thus, by the time it is diagnosed in patients, dysphagia may be well advanced. This progression, combined with the complex nature of the neurological control of feeding and swallowing, means that little is known about the neurological basis and time of onset of swallowing dysfunction in Parkinson's disease generally and in toxin-based models of the disease specifically. The purpose of this study was to begin to establish how oropharyngeal function is affected in rotenone models of Parkinson's disease. We demonstrate and characterize disruption of the different oropharyngeal behaviors that compose feeding and swallowing in an aggressive, fast-acting rotenone model of idiopathic PD [22], as a first attempt at documenting the effects of rotenone on swallowing and feeding physiology and function. Our null hypothesis for all variables is that there is no effect of rotenone treatment. Our alternative hypotheses are as follows:

- We hypothesize that range of motion during chewing of the tongue and jaw will increase in the dorsoventral axis and decrease in the rostrocaudal axis, as a result of dyskinesia affecting the complex palynal chewing stroke of rodents.
- We hypothesize that all measurements of duration in chewing (duration of chewing cycle, duration of jaw closing, duration of power stroke, time of tongue rostralmost position, and relative time of tongue and jaw movement) will increase due to bradykinesia caused by rotenone.
- We hypothesize that measurements of timing in swallowing (swallow onset delay, pharyngeal transit time, interswallow interval, and swallow rate) will increase following rotenone injections.

## 2. Methods

### 2.1. Rotenone Administrations Protocol

The methods followed Cannon et al. [22]. Rotenone crystals were diluted into a stock solution of DMSO and emulsified in Miglyol to produce an injectable solution. 12 adult Lewis rats (Charles River, Wilmington, MA) (6 females and 6 males, 14 weeks of age) were divided into two groups that received daily IP injections of the rotenone emulsification at either 2.75 mg/kg or 3 mg/kg. These doses represent the extremes of the range for which permanent loss of dopaminergic neurons, and associated decrease of motor function, was seen in the original study on which this work is based. As the study by Cannon et al. [22] documented phenotypic variation in the progression of parkinsonian symptoms under these two dose regimes, both doses were used here. Injections were administered daily until the rats showed significant weight loss, or significant debilitative behaviors (akinesia, poor grooming, and lack of feeding). Qualitative observation confirmed the development of parkinsonian symptoms (tail and limb rigidity, akinesia, and bradykinesia), as detailed in the results. All animal studies were approved by the NEOMED IACUC (protocol number 16-002) and followed NIH guidelines for the care and use of laboratory animals. In the high-dose group, animals were euthanized when they showed severely impaired locomotion and lack of feeding drive. In the low-dose group, animals were euthanized when the animals had lost 18% of their preinjection body mass.

### 2.2. Radio Opaque Marker Implantation and Videofluoroscopy

**Figure 1:**
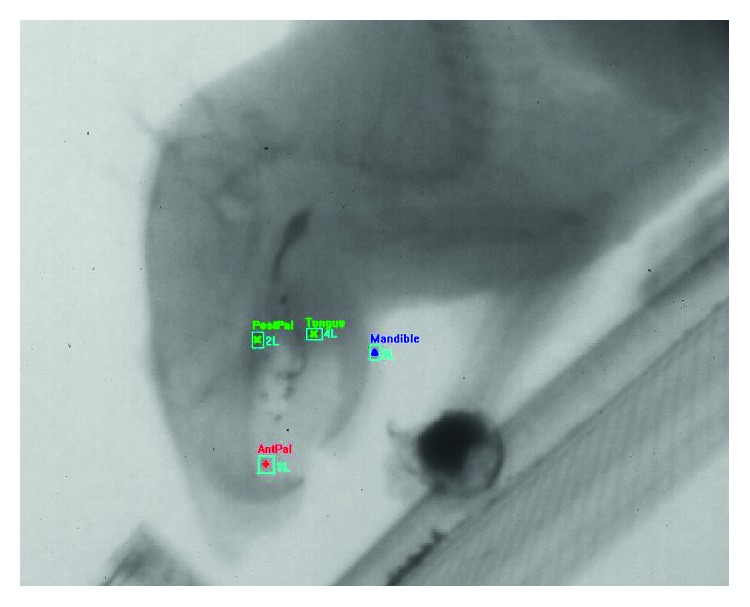
Videofluroscopic still from marker-tracking software, showing marker positions. PostPal: posterior palate; AntPal: anterior palate; Tongue: tongue; Mandible: mandible.

Prior to recording of behavior or injection of rotenone, the rats were trained to feed on breakfast cereal (Fruit Loops, Kellogg's, Battle Creek, MI) coated with radio opaque barium powder. Once the training was complete, the rats were anesthetized with isoflurane, and radio opaque markers were implanted into the tongue, palatal gingiva, and mandibular gingiva using a 22 gauge needle and a fine wire as a plunger (Figure 1). Rats were then recorded feeding using techniques adapted from pig [27, 28] and rodent [29] models of swallowing function. Feeding was recorded at 200 frames/second (MA: 4.0, kVp: 90) until 20–25 swallows had occurred in each session. X-rays were activated using a foot pedal when the animal began feeding and were turned off once twenty to twenty-five swallows had been counted on a monitor. This reduced radiation exposure and size of the recordings. Each recording was about 50 to 100 seconds long. The methods and dosage used for this study are standard and have been tested and validated in over 30 years of studies [30, 31]. No radiation damage to animals has been observed in that time. Two preinjection sessions were recorded and then once daily from the day of the first injection until the end of the experiment.

### 2.3. Data Collection

Feeding parameters were calculated for both chewing and swallowing. For chewing, multiple video segments comprising 5–7 consecutive chewing cycles (defined as cycles in which full molar occlusion could be seen in the video) were isolated from longer feeding videos, for a total of 15 to 20 chewing cycles per feeding bout. For each video segment, the position of the palatal, tongue, and mandibular markers was digitized using automated tracking software (ProAnalyst, Excitex, MA). These (*x*, *y*) coordinates were then scaled, rotated, and translated using the palate markers as reference points, following an established protocol [27]. This procedure removes elements of movement due to whole head movements and allows between sequence comparisons of the feeding kinematics. From these scaled, rotated, and translated data, we calculated a series of time and distance-based chewing variables (Table 1). As there were no markers in the pharynx, we were unable to extend the approach used for chewing to measure swallowing. Therefore, we measured the duration of several events directly from observation of the videos (Table 2). Summary statistics are reported in Supplementary Table 1. These variables were adapted from other studies of swallowing function in rodent models [32, 33]. Error studies were performed to assess the repeatability of these video scored events [34].

### 2.4. Statistical Analysis

Data were analyzed using linear mixed model multifactorial ANOVA with pre/postinjection dose, and their interaction as fixed factors, and individual as a random factor to account for interindividual variation. The units of analysis were the chewing cycle for all chewing variables. For pharyngeal transit time and swallow onset delay, the unit of analysis was the swallow. For swallow rate and interswallow interval, the unit of analysis was the feeding sequence. Although longitudinal data were collected, for this preliminary analysis, only the last time point of the rotenone pre/postinjection was included, as the focus was to document the presence of oropharyngeal dysfunction in this model. Significance level was set at 0.05. Where the interaction was significant, independent contrasts were used to test the specific effect of rotenone injection in high- and low-dose groups. For swallow onset delay, the inclusion of the extra factor (jaw cycle type) meant that there were insufficient degrees of freedom to perform a full model with pre/postinjection, dose, cycle type, and all potential interactions. Thus, high- and low-dose groups were analyzed separately, with pre/postinjection, jaw cycle type, and their interaction as factors in the model. All statistics and calculations were performed in R [35].

## 3. Results

### 3.1. Effects of Rotenone Injection on Weight and General Motor Behavior

In the high-dose group, weight loss was recorded in all rats within 48 h of the first injection and continued throughout the course of injection until the end of the experiments. Porphyrin buildup and poor pelage condition, indicating impaired grooming ability, were visible in most rats from day three. Reduced to absent feeding drive is manifested from day 4. Hypokinesis, manifesting as limited motion around the cage and limited use of hindlimbs (resulting in a twisted posture when feeding), was evident from day 4. Rats in this group reached complete immobility with inability to locomote or maintain posture by day five.

In the low-dose group, weight loss began after the first injection, and by day 7 postinjection had all lost 18% of the initial body weight, the IACUC approved the end point of the study. In this group, tail rigidity, mild postural changes, and reduction in pelage condition were noted from day four. Phenotypic progression was much less than that in the high-dose group and did not progress to complete immobility.

### 3.2. Effects of Rotenone on Chewing and Swallowing Variables

**Figure 2:**
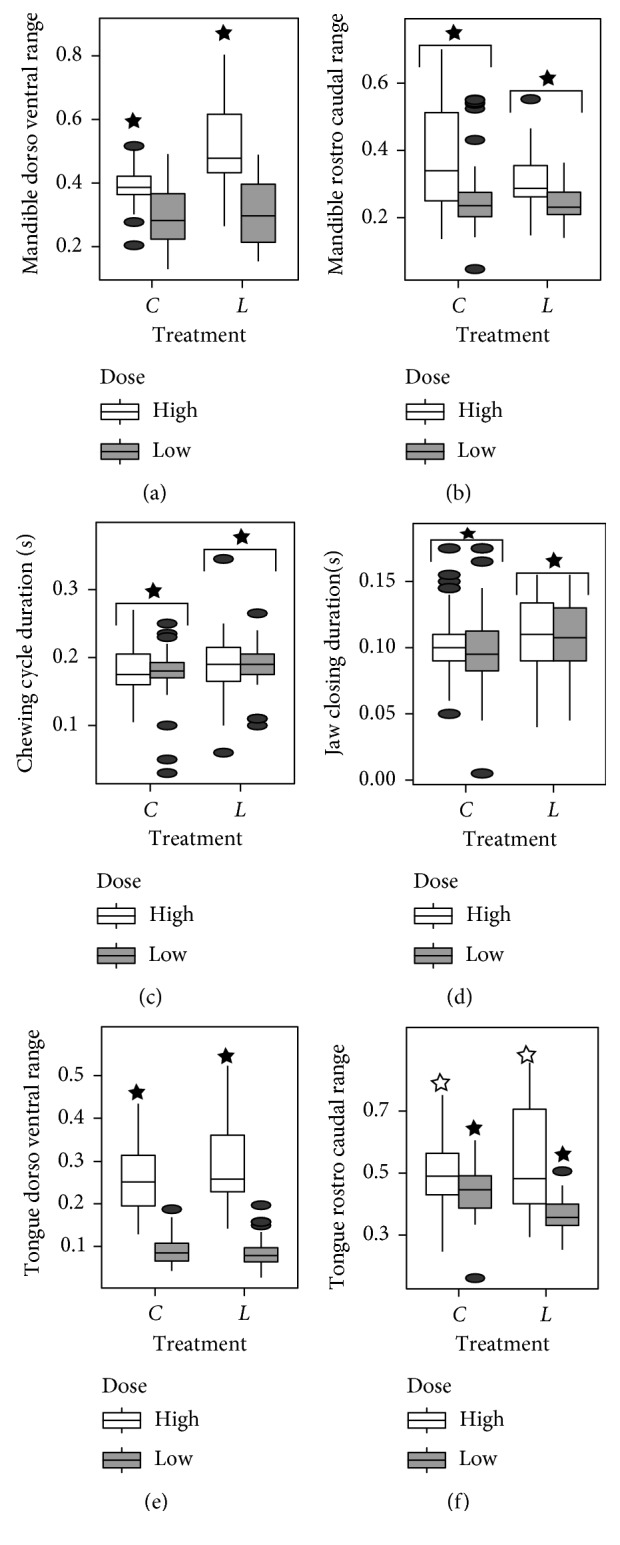
Boxplots of the chewing variables for which significant injection or injection-dose interactions were found. Stars indicate pairwise differences that were significant (*p* < 0.005). *n*=188 chew cycles. C: preinjection measurements and L: last day postinjection measurements.

**Figure 3:**
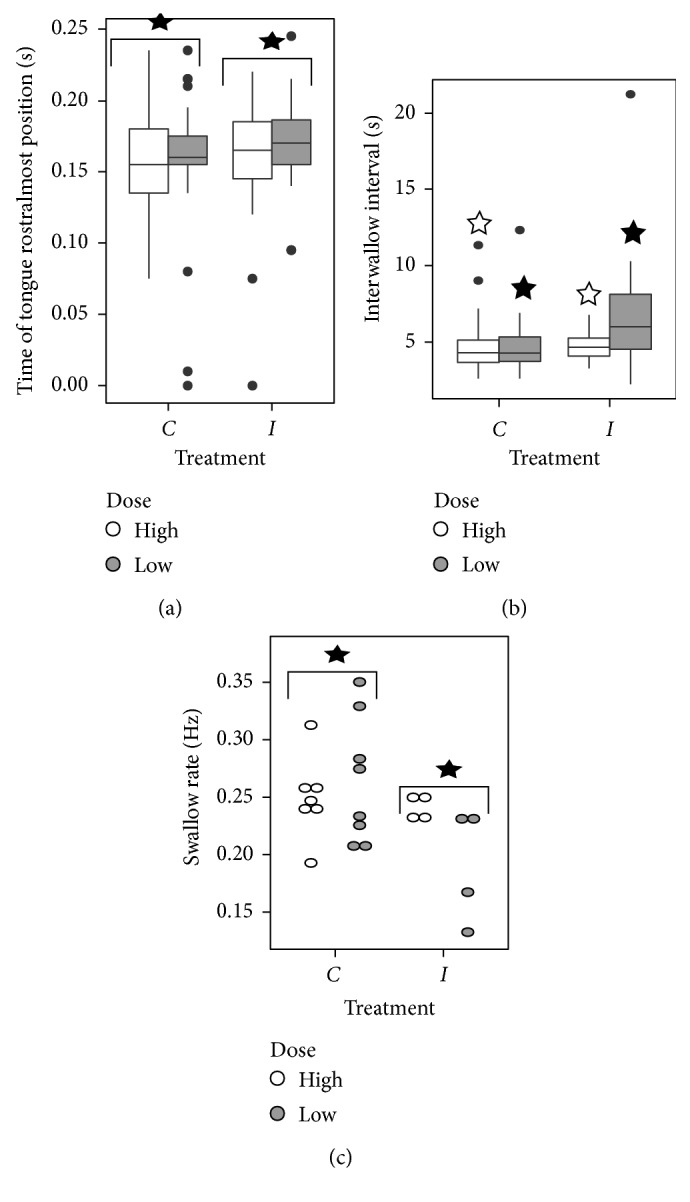
Boxplot and doplot of tongue and swallowing variables for which significant injection or injection-dose interactions were found. Stars indicate significant pairwise differences (*p* < 0.05). *n*=188 chews (time of tongue rostralmost position). *n*=199 swallows (interswallow interval). *n*=20 sequences (swallow rate). C: preinjection measurements, L: last day postinjection measurements.

Statistical results for the chewing variables are summarized in Supplementary Table 2(a). A significant effect of injection with rotenone, regardless of dose level, was found for rostrocaudal mandibular range of motion (*F*(1,184) = 16.49, *p* < 0.001), duration of chewing cycle (*F*(1,184) = 5.25, *p*=0.023), duration of jaw closing (*F*(1,183) = 6.95, *p*=0.009), and time of tongue rostralmost movement (*F*(1,183) = 4.67, *p*=0.032). The hypothesis of longer duration of movements posttreatment is supported for chewing cycle duration, jaw closing duration, and time of rostralmost tongue position. In the high-dose rats, there was an effect of injection on dorsoventral mandibular range of motion (*p* < 0.001), tongue dorsoventral range of motion (*p*=0.049), and tongue rostrocaudal range of motion (*p* < 0.001). In the low-dose rats, there was an effect of injection on tongue rostrocaudal range of motion (*p* < 0.001) (Figure 2). Effects of injection on swallowing variables were less pervasive than that on chewing variables (Supplementary Tables 2(b) and 2(c)). There was an effect of injection on the rate of swallowing (*F*(1,13) = 5.67, *p*=0.033). In both the low- and high-dose injection groups, interswallow interval was longer (low dose *p* < 0.001, high dose *p* < 0.001). In the low-dose group, there was a significant effect of the interaction of injection and jaw cycle type on the delay between start of the previous jaw cycle and start of the swallow (*F*(1,55) = 7.94, *p*=0.007). However, there is no significant difference between swallow start delay pre- and postinjection in either ingestion or chewing cycles (Figure 3).

## 4. Discussion

The null hypothesis of no effect could not be rejected for duration of power stroke, relative timing of tongue and jaw movement, pharyngeal transit time, and swallow onset delay. For the tongue and jaw range of motion in chewing, the alternative hypothesis of increased dorsoventral and decreased rostrocaudal range of motion was supported. For timing variables where the null hypothesis was rejected (chewing cycle duration, jaw closing time, time of tongue rostralmost position, interswallow interval, and chewing rate), the alternative hypothesis that durations increased, indicating a slowing down of movement, was supported.

The most consistent perturbations are found in chewing movement speed and range of motion changing. Chewing is affected in parkinsonian patients [36–38]. In our study, pharyngeal swallowing duration is not affected and swallowing rate decreases. This is informative neurologically and anatomically, as the embryonic origins and neural systems controlling the jaw and the pharynx are distinct [39–41]. Although these results suggest that dysphagia results from chewing deficits leading to inadequate bolus formation prior to swallow, previous work cautions against this interpretation. Chewing responds to L-dopa [36], yet this treatment does not improve dysphagia outcomes in PD patients in studies using both qualitative assessments and videofluoroscopic swallow studies [14].

Although central nervous system degeneration hypotheses could explain these patterns, peripheral neural and even muscular changes in patients with Parkinson's disease, affecting the function of nerves and muscles outside the central nervous system [42, 43], may also lie behind some of these differences.

These preliminary findings support further research into the rotenone model of Parkinson's disease to study dysphagia and oropharyngeal dysfunction in Parkinson's disease. If verified in models of Parkinson's disease that more closely reflect the clinical progression of the disease, such research may indicate new avenues for neurological and behavioral research into these debilitating conditions. General slowing down of the entire feeding process, changes in chewing kinematics, and limited effect on the pharyngeal transit time of swallowing are consistent with results from the Pink1 rat model of genetic PD [25]. We deliberately chose a fast-acting, aggressive model, yet we found differences between the two groups. Some of these may be due to the fact that the high-dose group did not receive injections for as long (the high-dose group on average survived three days of injections, the low-dose group seven). With the accelerated toxicity seen in the high-dose group, not all musculoskeletal functions may be impaired to the same degree. Given the known role of environmental toxicant exposure in Parkinson's disease, and the variability of such exposure, dosage dependency may be clinically important regarding underlying variability of Parkinson's [1, 44]. Further studies are necessary to explicitly and systematically investigate dose-dependent effects. The variation in the presence and severity of dysphagia in Parkinson's disease [20] may reflect differences in the etiology of the disease, some of which may be due to duration and severity of environmental toxin exposure, which we can model in animal systems.

This study is a preliminary attempt at documenting oropharyngeal dysfunction in a well-characterized toxicological model of Parkinson's disease. We chose to use an aggressive, fast-acting model of rotenone-induced Parkinson's disease, which limits the immediate applicability to feeding dysfunction in the human disease by omitting the gradual, progressive nature of Parkinson's disease. Future work with newer, more gradual models of rotenone-induced neurodegeneration will bridge this gap. We acknowledge limitations of this study. The study was preliminary and focused on establishing changes in oropharyngeal function in a well-validated, reliable model of pesticide-induced Parkinson's disease. As such, important data, including quantitative measurements of limb discoordination and neurodegeneration, were not included. Future studies will incorporate a more complete view of behavioral and neural degradation, including neuron damage in regions outside the nigrostriatal complex. As a result, some caution is warranted in overinterpreting these results with regard to their significance for clinical cases of Parkinson's-induced dysphagia. We only compared the end points reached by each animal relative to preinjection control recordings, so there are no results on progression. Owing to individual variation in response to toxicity and difficulties in obtaining behavioral data towards the end of the experiment, the individual animal end points vary by up to 48 hours within each dosage group. The large variances seen in our measurements may reflect different degrees of impairment at the date of last recording. Some animals for which we did not obtain later recordings may have still been able to feed but have been unwilling due to suppressed feeding drive. Thus we have a weak control for similarity in the degree of impairment. A major challenge of adopting rodent models of neurological disease for the study of dysphagia lies in the highly derived feeding behavior and anatomy of rodents, such that they rarely aspirate (take food into the airway). Thus, the main diagnostic criterion of clinical dysphagia in human patients is not transferable to rodents. However, general changes in oropharyngeal function in rodents are informative about neurological mechanisms specific to feeding that are affected in Parkinson's disease and affect the general quality of life of patients in the broader context of eating as a complex motor behavior. The usefulness of rodent models for studying these diseases is spurring research into the development of rodent-specific measures of feeding efficiency that are explicitly tied to measurable physiological changes in neuromotor function [32, 33, 45].

## Figures and Tables

**Table 1:** Variables measured in this study: chewing variables.

Variable	Description
Mandible: dorsoventral range	Within a chewing cycle, maximum distance travelled in the dorsoventral (*y*) axis by the mandibular marker.
Mandible: rostrocaudal range	Within a chewing cycle, maximum distance travelled in the rostrocaudal (*x*) axis by the mandibular marker.
Duration of chewing cycle	Time elapsed from maximum gape to maximum gape.
Duration of jaw closing	Time elapsed from maximum gape to mimimum gape within a cycle.
Duration of power stroke	Time from minimum gape to rostralmost position of the mandible.
Tongue: dorsoventral range	Within a chewing cycle, maximum distance travelled in the dorsoventral (*y*) axis by the tongue marker.
Tongue: rostrocaudal range	Within a chewing cycle, maximum distance travelled in the rostrocaudal (*x*) axis by the tongue marker.
Time of tongue rostralmost position	Time from beginning of chewing cycle to tongue reaching its rostralmost position.
Relative timing of tongue and jaw	Time from the mandible reaching occlusion to the tongue reaching its rostralmost position.

**Table 2:** Variables measured in this study: swallowing variables.

Variable	Description
Pharyngeal transit time	Time from first frame of movement of the bolus into the oropharynx to when the tail of the bolus passes the level of cervical vertebra 4.
Interswallow interval	Time between the onset of two consecutive swallows.
Swallow rate	Ratio of number of swallows to duration of feeding bout.
Swallow onset delay	Time between maximum gape of previous jaw cycle and onset of swallow inserted within that cycle. Categorised by jaw cycle type (chewing or biting/ingestion).

## Supplementary Materials

Supplementary Materials Supplementary Table 1: summary statistics. Supplementary Table 2: results of all statistical tests and complete results of linear mixed model analysis of variance for all variables. Bold values indicate significance.
