# Supplementary material for: Evidence of Oropharyngeal Dysfunction in Feeding in the Rat Rotenone Model of Parkinson's Disease
Source: Parkinsons Dis. 2018 Mar 11;2018:6537072. doi: 10.1155/2018/6537072 (PMC5866867; doi:10.1155/2018/6537072)
Supplement: Supplementary Materials — Supplementary Table 1: summary statistics. Supplementary Table 2: results of all statistical tests and complete results of linear mixed model analysis of variance for all variables. Bold values indicate significance. [file 6537072.f1.docx]

Supplementary data

Supplemental table 1: summary statistics

| Variable | High dose | | | | | | Low dose | | | | | | |
| --- | --- | --- | --- | --- | --- | --- | --- | --- | --- | --- | --- | --- | --- |
|  | Control | | | Post injection | | | Control | | | Post Injection | | | |
|  | Count | Mean | Sd | Count | Mean | SE | Count | Mean | SE | | Count | Mean | SE |
| Mandible: dorso ventral range | 41 | 0.39 | 0.06 | 42 | 0.52 | 0.13 | 55 | 0.30 | 0.09 | | 53 | 0.31 | 0.1 |
| Mandible: rostro caudal range | 41 | 0.38 | 0.15 | 42 | 0.31 | 0.09 | 55 | 0.26 | 0.11 | | 53 | 0.24 | 0.05 |
| Duration of chewing cycle | 41 | 0.18 | 0.03 | 42 | 0.19 | 0.05 | 55 | 0.18 | 0.03 | | 53 | 0.19 | 0.03 |
| Duration of jaw closing | 41 | 0.1 | 0.03 | 42 | 0.11 | 0.03 | 55 | 0.1 | 0.03 | | 52 | 0.11 | 0.03 |
| Duration of power stroke | 41 | 0.02 | 0.01 | 42 | 0.02 | 0.01 | 55 | 0.02 | 0.02 | | 52 | 0.02 | 0.01 |
| Tongue: dorso ventral range | 41 | 0.26 | 0.08 | 42 | 0.3 | 0.1 | 55 | 0.09 | 0.03 | | 53 | 0.08 | 0.03 |
| Tongue: rostro caudal range | 41 | 0.049 | 0.12 | 42 | 0.54 | 0.17 | 55 | 0.44 | 0.08 | | 53 | 0.36 | 0.05 |
| Time of tongue rostralmost position | 41 | 0.16 | 0.03 | 42 | 0.16 | 0.04 | 55 | 0.16 | 0.04 | | 52 | 0.17 | 0.03 |
| Relative time of tongue and jaw | 41 | 0.06 | 0.01 | 42 | 0.06 | 0.01 | 55 | 0.06 | 0.02 | | 52 | 0.06 | 0.02 |
| Pharyngeal transit time | 54 | 0.19 | 0.06 | 26 | 0.2 | 0.05 | 61 | 0.2 | 0.08 | | 38 | 0.21 | 0.05 |
| Inter swallow interval | 54 | 4.59 | 1.44 | 26 | 4.73 | 0.79 | 61 | 4.57 | 1.42 | | 38 | 6.65 | 3.07 |
| Swallow rate | 7 | 0.25 | 0.04 | 4 | 0.24 | 0.01 | 8 | 0.26 | 0.05 | | 4 | 0.19 | 0.05 |

| Variable | Ingestion cycle | | | | | | Chew cycle | | | | | | |
| --- | --- | --- | --- | --- | --- | --- | --- | --- | --- | --- | --- | --- | --- |
|  | Control | | | Post injection | | | Control | | | Post Injection | | | |
|  | Count | Mean | Sd | Count | Mean | SE | Count | Mean | SE | | Count | Mean | SE |
| Swallow start delay: high dose | 11 | 0.07 | 0.02 | 13 | 0.12 | 0.08 | 21 | 0.11 | 0.04 | | 17 | 0.1 | 0.04 |
| Swallow start delay: low dose | 8 | 0.07 | 0.01 | 5 | 0.08 | 0.02 | 5 | 0.12 | 0.05 | | 5 | 0.1 | 0.01 |

Supplemental table 2: complete results of linear mixed model analysis of variance for all variables. **Bold** values indicate significance.

2a: results for chewing variables

| Variable | Pre/post injection | Dose | Pre/post injection:dose | Within dose high | Within dose low |
| --- | --- | --- | --- | --- | --- |
| Mandible: dorso ventral range | **F(1,184)=27.49, p<0.001** | F(1,3)=5.165, p=0.108 | **F(1,184)=36.6 p<0.001** | p**<0.001** | p=0.996 |
| Mandible: rostro caudal range | **F(1,184)=69.67, p<0.001** | F(1,3)=1.9, p=0.262 | F(1,184)=2.78 p=0.09 | NA | NA |
| Duration of chewing cycle | **F(1,184)=5.25**  **p=0.023** | F(1,3)=0.02, p=0.9 | F(1,184)=0.19, p=0.663 | NA | NA |
| Duration of jaw closing | **F(1,183)= 6.95, p=0.009** | F(1,3)=0.03, p=0.871 | F(1,183)=0.51, p=0.475 | NA | NA |
| Duration of power stroke | F(1,183)=1.917, p=0.168 | F(1,3)=0.69, p=0.468 | F(1,183)=0.45, p=0.504 | NA | NA |
| Tongue: dorso ventral range | F(1,184)=2.05, p=0.154 | **F(1,3)=11.8, p=0.041** | **F(1,184)=14.15, p<0.001** | **P<0.001** | P=0.288 |
| Tongue rostro caudal range | **F(1,184)=4.38, p=0.038** | F(1,3)=2.23, p=0.232 | **F(1,184)=29.81, p<0.001** | **P<0.001** | **P<0.001** |
| Time of tongue rostralmost position | **F(1,183)=4.674, p=0.032** | F(1,3)=0.29, p=0.629 | F(1,183)=0.59, p=0.445 | NA | NA |
| Relative timing of tongue and jaw | F(1,183)=0.06, p=0.809 | F(1,3)=3.38, p=0.163 | F(1,183)=0.02, p=0.886 | NA | NA |

2b: results for swallowing variables

| Variable | Pre/post injection | Dose | Pre/post injection:dose | Within dose high | Within dose low |
| --- | --- | --- | --- | --- | --- |
| Pharyngeal transit time | F(1,192)=0.248, p=0.619 | F(1,6)=0.01, p=0.936 | F(1, 192)=0.06, p=0.812 | NA | NA |
| Inter swallow interval | **F(1,169)=21.32, p<0.001** | F(1,6)=1.66, p=246 | **F(1,169)=11.39, p<0.001** | **P<0.001** | **P<0.001** |
| Swallow rate | **F(1,13)= 5.672, p=0.033** | F(1,6)=0.15, p=0.709 | F(1,13)=3.47 p=0.085 | NA | NA |

2c: results for relative timing of swallowing to chewing

| Variable | Pre/post injection | Cycle | Pre/post injection:cycle | Within Chew | Within bite |
| --- | --- | --- | --- | --- | --- |
| Swallow delay low | F(1,55)=1.44, p=0.235 | F(1,55)=1, p=0.32 | **F(1,55)=7.94, p=0.0067** | P=0.695 | P=0.830 |
| Swallow delay high | F(1,18)=0.014, p=0.907 | F(1,18)=12.653,  P=0.0021 | F(1,18)=1.524, p=0.232 | NA | NA |
